# Perspective of Patients With Metastatic Breast Cancer on Electronic Access to Scan Results: Mixed Methods Study

**DOI:** 10.2196/15723

**Published:** 2020-02-10

**Authors:** Christina Baun, Marianne Vogsen, Marie Konge Nielsen, Poul Flemming Høilund-Carlsen, Malene Grubbe Hildebrandt

**Affiliations:** 1 Department of Nuclear Medicine Odense University Hospital Odense Denmark; 2 OPEN, Odense Patient data Explorative Network Odense University Hospital Odense Denmark; 3 PREMIO, Centre for Personalized Response Monitoring in Oncology Odense University Hospital Odense Denmark; 4 Department of Oncology Odense University Hospital Odense Denmark; 5 Research Unit on User Perspectives Department of Public Health University of Southern Denmark Odense Denmark; 6 Department of Clinical Research University of Southern Denmark Odense Denmark; 7 CIMT, Centre for Innovative Medical Technology Odense University Hospital Odense Denmark

**Keywords:** patient accessible electronic health record, electronic health records, patient access to records, scan result, breast cancer, patient perspective, breast neoplasms

## Abstract

**Background:**

Patient-accessible electronic health records give patients quick and easy access to their health care data, enabling them to view their test results online prior to a clinic visit. Hospital reports can be difficult for patients to understand, however, and can lead to unnecessary anxiety.

**Objective:**

We aimed to investigate the attitudes and experiences of Danish patients with metastatic breast cancer in using electronic health records to view their own scan results.

**Methods:**

We conducted a prospective mixed methods study in a sequential design at our institution during 2018. Participants were women with metastatic breast cancer who were having scans every 3 months (combined positron emission tomography and computed tomography or computed tomography alone) to monitor treatment effects. Participants first received an online questionnaire about their knowledge and use of online access to scan results. We then conducted semistructured interviews with 4 women who used the online access to view their scan results.

**Results:**

A total of 46 patients received the questionnaire (median age 66, SD 11.8, range 34-84 years). Of these women, 38 (83%) completed the survey (median age 69, SD 10.7, range 42-84 years). Most patients (34/38) were aware of the opportunity to access their reports online, but only 40% (15/38) used this access to read their scan results. Barriers to online access were (1) anxiety over reading the scan results in the absence of clinician support, and (2) a preference to receive all disease information at their next hospital appointment. The patients who read their scan result found that facilitators were greater transparency and empowerment, and barriers were the consequences of reading bad news, the feeling of dilemma about the access, and the medical terminology.

**Conclusions:**

Patients with metastatic breast cancer generally had a positive attitude toward electronic access to their scan results, and those who used this opportunity played a greater participatory role in their disease and its management. Others described the potential distress this opportunity caused. The study findings suggest that immediate online access to scan results should be available to patients, but it needs a support function alongside that ensures optimal patient care.

## Introduction

### Patient-Accessible Electronic Health Records

Health care has become more patient centered over the last decade and, together with the digitization era, this has resulted in implementation of patient-accessible electronic health records (PAEHRs) [1,2]. The PAEHR is an important part of the effort to include the patient as an active player in their own health care [3,4]. Giving patients full access to their own health data increases transparency and gives the patient greater insight into individual health conditions and treatment plans. This can facilitate the communication between patient and health care professionals and thereby encourage more active patient participation in own health care [5]. Access to adequate and relevant information is a step toward greater patient empowerment, including greater patient participation in individual health decisions and a reduced sense of inequality for the patient [6].

Patients with chronic diseases tend to use online health information more frequently [7], especially in relation to test results [4,8]. When patients access test results on their own, however, the detailed information from a health care professional is lacking, and the report may be difficult for the patient to understand [8,9]. This may cause the patient to misinterpret the results, leading to unnecessary distress or anxiety [3,10].

The Danish national PAEHR, sundhed.dk*,* was launched in 2003 [11]. sundhed.dk is the official portal for the public Danish health care services and enables citizens and health care professionals to find information and communicate with each other. The portal facilitates patient-centered digital services that provide access to and information about the Danish health care services, including all clinical domains. sundhed.dk gives patients fast and easy access to their full medical record and test results in a secure and confidential way [12]. Patient access to imaging and test results was initially delayed by several days, but this has now improved so that patients can access their results immediately after a test or examination has been performed.

Patient access to online health records is not available in all countries, and to our knowledge only limited data have been reported on patients’ experiences with online access. Recommendations and case studies have been published in the effort to optimize the use and experience of PAEHRs, especially regarding the level and timing of access to test results, but this is mostly from the perspective of the health care system [3,13]. We lack information about the different settings for PAEHRs and, even more importantly, about the patient perspective.

Patients with chronic disease often have regular diagnostic imaging to evaluate the effect of treatment. The scan results can be crucial for decisions about future treatment and are thus very important for the individual patient, for example with metastatic breast cancer [14]. This patient group is assumed to have a strong incentive to access their scan results online, and their perspectives and experiences can contribute to our knowledge about online access to patient records and the potential advantages and disadvantages.

### Objective

We aimed to increase knowledge about patients’ experiences of online access to scan results and to identify any unforeseen issues as part of the effort to optimize work practices as experienced by the patient. This study prospectively investigated how women with metastatic breast cancer use the Danish electronic health record system to read their scan results and explored the women’s attitudes toward and experiences with this patient access.

## Methods

### Study Design

We carried out an explorative mixed methods, single-center study involving patients with metastatic breast cancer prospectively from January to May 2018 at the Department of Nuclear Medicine, Odense University Hospital, Odense, Denmark. We combined quantitative and qualitative methods in a sequential design. The women first received an electronic questionnaire about their knowledge and use of the online health record. We then conducted individual semistructured interviews with 4 of the women, aiming to elaborate on the findings from the questionnaire and to obtain a more individual perspective of the women’s attitudes toward and experiences with online access to their scan results.

### Patient Selection

The 53 white women with metastatic breast cancer who were invited to participate in this study were already enrolled in a larger retrospective diagnostic study at the department, analyzing the use of computed tomography (CT) and positron emission tomography with computed tomography (PET/CT) for response monitoring in metastatic breast cancer. The women were scanned with either CT at the Department of Radiology or PET/CT at the Department of Nuclear Medicine every third month to monitor the effect of ongoing oncological treatment as part of daily clinical routine; hence, no intervention was performed. All women had accepted enrollment in the retrospective study and given permission for further contact regarding potential other research projects, which was a main reason for inviting this patient group to participate in this substudy. Previous experience with the health portal was not a criterion. Therefore, in the survey we included first-time users, experienced users, and women who had never used the portal. Exclusion criteria were women who were not regularly monitored with either CT or PET/CT, and patients who did not use their secure digital post system to receive information from health authorities.

### Ethics and Approval

The study was conducted in compliance with the Declaration of Helsinki and approved by the Danish Data Protection Agency. We obtained written informed consent from all participants prior to study entry, and anonymized and handled personal data according to current legislation.

### The Questionnaire

The objective of the questionnaire was to investigate the patients’ use of the PAEHR and their attitudes toward the health portal and access to scan results. We conducted an exploratory interview with 1 breast cancer patient initially to uncover themes and relevant issues. The questionnaire was developed iteratively by a collaborative team comprising a research radiographer (CB), a specialist in nuclear medicine (MGH), 2 nuclear medicine technicians, a secretary, an oncology physician (MV), and 2 patient representatives who had previously undergone treatment for primary breast cancer. The patient representatives helped to design and formulate the questionnaire, optimize the language, and improve relevance of the questions and response options. The questionnaire underwent several pilot tests before the main survey.

In January 2018, the 53 women received an information letter by email through the secure digital post system. The letter included an embedded URL that linked patients directly to the questionnaire. We sent a follow-up email after 1 week to those who had not replied and closed the survey after 30 days.

The digital questionnaire was interactively designed so that it adapted to the individual respondent’s answers. The number of questions ranged from 17 to 22 depending on the individual’s experience with the PAEHR. The survey had forced multiple choice questions and took approximately 15 minutes to complete.

The first part of the survey comprised (1) 7 closed-ended demographic questions, (2) 4 closed-ended questions about the patient’s knowledge and use of the Danish PAEHR, including whether the patient had been informed about the health portal by a health care professional, and (3) a series of open-ended or partly open-ended questions on attitudes toward and experiences with the PAEHR.

If the patient had never used the online access they were asked about the underlying reasons and attitudes for this. Nonusers of the PAEHR were informed about the recent change to remove the delay in access to test results and were asked about their attitudes toward this and possible benefits and drawbacks. The questions were partly open ended, with 5 to 6 response options and the possibility to supplement their response with a comment.

Users of the PAEHR were asked partly open-ended questions about their reasons for using the PAEHR and what they experienced as benefits or challenges. Several response options were provided as well as a comment field. Active users were then asked how often and under what circumstances they used the online access, and which aspect of the portal they used, such as their medical record, test results, or medication list. The women were also asked whether they shared or viewed their online medical information with a family member or friend and the reasons for this.

Women who did read their test results online were asked (1) whether they had experienced a need to contact a health care professional after reading their test results and whether they had acted on this, (2) whether they had experienced any changes or developments in the health portal during their time of use (to see if they had noticed the removal of the delay in test results), and (3) after a short explanation of the change to remove the delay in test results, their attitudes toward and experiences with immediate access to test results.

An open comment field at the end of the questionnaire invited all respondents to supplement their responses with any other relevant issues or comments. Finally, women who used the PAEHR to read their scan results were invited to participate in a follow-up interview about their experiences with the PAEHR. The women who agreed to participate were asked to give their contact information and to indicate their preferred mode of communication.

### Individual Interviews

We designed a semistructured interview guide based on the survey results and focusing on the patients’ experiences of potential benefits and drawbacks of having online access to their scan results. The interview guide consisted of 6 major themes that framed the overall interview: (1) knowledge and use of online health care options, (2) experience with and attitudes toward online access to diagnostic results in sundhed.dk, (3) experience with and attitudes toward immediate access to scan results, (4) the patient role, (5) the role of health care professionals, and (6) trust in the health care system. The inclusion criteria for the interview were women who completed the questionnaire and used sundhed.dk to access their scan results. Of those who agreed to participate, we selected 4 women of different ages, education level, cohabitation status, and time of metastatic cancer recurrence. Each informant was contacted by phone or email according to their preference, and an interview date was arranged.

The individual interviews were conducted face-to-face by the first author (CB) in February and March 2018. At the patients’ request, 3 interviews were held in the informants’ own home and the fourth at the Department of Nuclear Medicine. The interviews lasted approximately 60 minutes and were audio recorded. Audio files of the 4 interviews were imported into a REDCap database (REDCap Consortium) and exported to NVivo 11.4.1 pro (Windows version; QSR International). Each interview was transcribed the day it was conducted. Transcription and data analysis were performed in NVivo by the first author (CB).

### Analysis of Quantitative Data

The questionnaire data were imported into a REDCap database and exported to Stata (M*P* 14.0; StataCorp LLC). We supplemented descriptive statistics with figures and graphs created on REDCap and in Excel 2010 (Microsoft Corporation). We used nonparametric Wilcoxon rank sum test with a significance level of 5% for comparison of differences between groups of users and nonusers of the PAEHR. Open-ended comments were collected and used to develop the interview guide and are quoted here with respondent number.

### Analysis of Qualitative Data

The data from each interview were analyzed and thematically coded in 4 steps, using interpretative phenomenological analysis developed by Smith [15,16]. These step were as follows: (1) in-depth and iterative review of the transcribed data, including highlighting of distinctive phrases and writing notes, (2) conceptualization of emergent themes and memos from each interview to develop a codebook frame, (3) hierarchical clustering of the emergent themes from each transcript, under a descriptive label, and (4) selection of the major relevant themes and representative quotes. The quotes included in the Results section are identified by informant number.

Within the overall frames of the interview, the analysis identified 13 major themes. Of these themes, 6 themes were not directly related to the topic of this study (the role of cancer, inequality, hope and anxiety, information loss, attitudes of health care professionals, paradigm shift, and the health portal in general). Therefore, we report here on only 7 of the major themes within 2 frames from the interview, as follows.

#### The Women’s Attitudes Toward and Experiences With Online Access to Scan Results

Themes in this frame were (1) greater transparency and patient empowerment, (2) consequences of “bad news,” (3) creation of a dilemma, and (4) medical language.

#### The Women’s Attitudes Toward and Experiences With Immediate Online Access to Scan Results

Themes in this frame were (5) differences according to scan type, (6) increased need for contact with the oncology team, and (7) their own responsibility.

## Results

### Participant Characteristics

We refer to participants in the survey as *respondents* and those in the interviews as *informants*.

**Figure 1:**
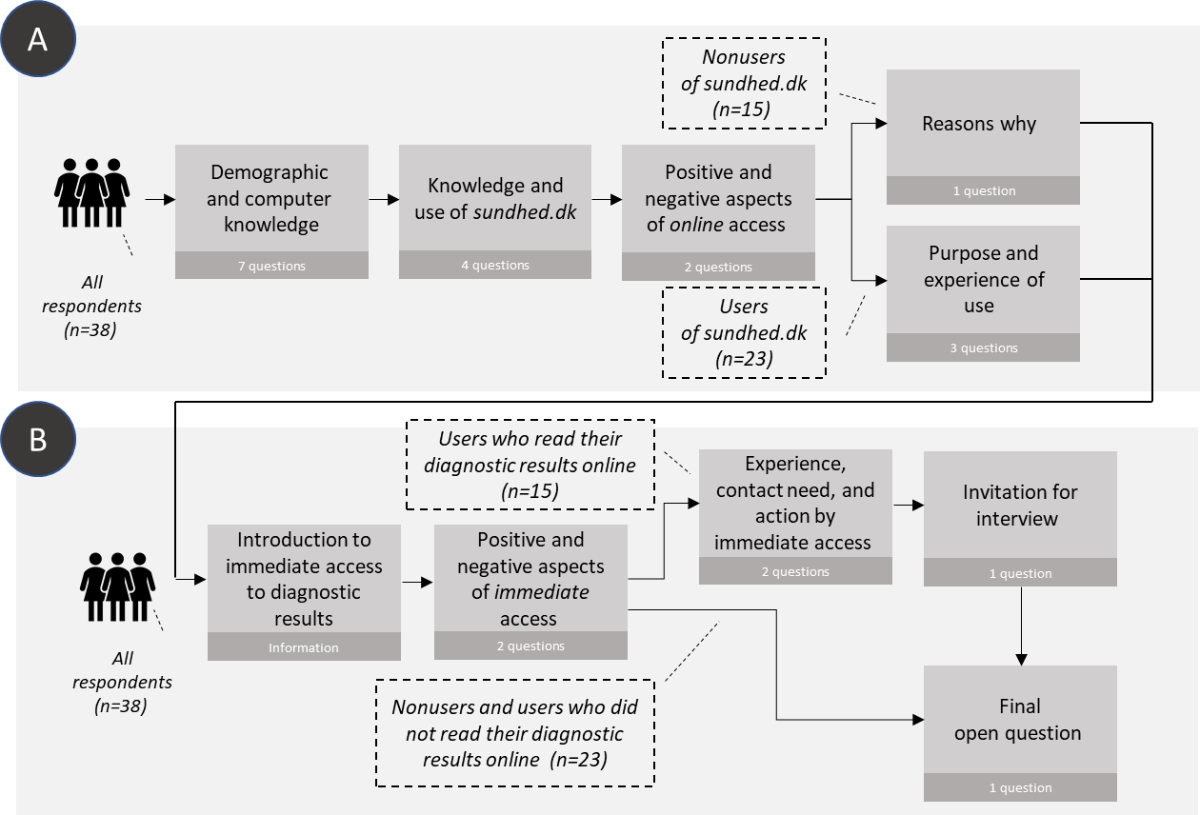
Overview of the online questionnaire, illustrating the interactive nature of the questionnaire and showing which respondent groups were given the different questions in each section. Section A primarily addressed attitudes toward and experiences with the patient-accessible electronic health records and section B addressed immediate access to scan results. All 38 respondents were asked about their attitudes toward online access and immediate online access regardless of previous use and experience.

Of the 53 invited patients, we excluded 7 women due to no active use of their secure digital post system, and thus 46 women received the questionnaire (median age 66, SD 11.8, range 34-84 years). Of these, 38 replied (response rate of 83%). Figure 1 illustrates the different sections of the interactive questionnaire and Table 1 summarizes sample characteristics.

**Table 1:** Characteristics of 38 white women with metastatic breast cancer who completed the online questionnaire.

Characteristics		Values	
**Age (years)**			
	Median (SD)		69 (10.7)
	Range		42-84
**Time since recurrence diagnosis (years)**			
	Median (SD)		1.3 (1.9)
	Range		0.7-8.1
**Highest education level, n (%)**			
	Primary school		7 (18)
	Trade, technical, or vocational training		8 (21)
	High school		1 (3)
	Intermediate degree (<3 years)		10 (26)
	Bachelor’s degree (3-5 years)		9 (24)
	Master’s degree (≥5 years)		3 (8)
**Household status, n (%)**			
	Living with partner or family		25 (66)
	Living alone		13 (34)
**Speaking and writing fluent Danish, n (%)**			
	Yes		37 (97)
	No		1 (3)
**Regular use of a computer, n (%)**			
	Daily		29 (76)
	Weekly		7 (18)
	Seldom		1 (3)
	Never		1 (3)

Only 2 respondents did not use a computer on a daily or weekly basis. The survey data included 11 open-ended comments from 10 respondents. The 8 nonresponders had a median age of 55 (SD 15.2) years (range 34-82 years) and median time since recurrence of 4.0 (SD 1.6) years (range 0.0-5.6 years), and no further basic information was available for this group.

### Knowledge and Use of the Danish Patient-Accessible Electronic Health Records

Of the 38 respondents, 36 (95%) knew about the PAEHR and 34 (90%) were aware that they could read their test results online. One-third (12/38, 32%) had received information about the PAEHR from a health professional. Of the 23 active users of sundhed.dk, most respondents accessed the electronic health record to read their medical file (22/23, 96%), which included notes from the physician and nurse, or their scan results (15/23, 65%). The respondents also read their laboratory results (11/23, 48%), medication list (9/23, 39%), and other online data, which included information regarding dental care, physiotherapy, and rehabilitation. Multiple answers were possible for this question.

Of the respondents (15/38, 40%) who viewed their scan results online, 12 agreed to participate in the interviews and 60% (9/15) shared the information with a spouse or partner and to a lesser degree with their children. The purpose for sharing the information was to better understand the report and for psychological support.

Table 2 shows demographic data for the 4 interview informants.

Of the 4 women, 3 reported that they regularly used the online access and always read their scan results as soon as these were released. They also used the online access to read their medical record, to see their blood test results, and to be prepared and well informed prior to their appointment at the hospital.

**Table 2:** Overview of the 4 women selected for individual interview.

Informant	Age (years)	Time since metastasis (years)	Marital status	Educational level	Routine scanning
1	Early 40s	3	Married, 2 children living at home	Intermediate degree	PET^a^/CT^b^
2	Late 50s	6.5	Living alone	Trade or technical training	CT
3	Late 60s	1	Living alone	Master’s degree	CT and PET/CT
4	Late 70s	1	Married	Intermediate degree	CT and PET/CT

^a^PET: positron emission tomography.

^b^CT: computed tomography.

The 15 women who had never used the online access to read their scan results stated 2 primary reasons in the questionnaire: either they did not want to view their results in the absence of clinician interpretation and support, or they expected to receive all the necessary information about their disease status at their next hospital appointment.

The 23 women using the PAEHR had higher educational levels (10/23, 44% with a bachelor’s or master’s degree) than the 15 nonusers (2/15,13%; *P*=.05), but did not differ in age (users: median 68, SD 11.2, range 42-84 years; nonusers: median 69, SD 10.2, range 48-80 years).

### Attitudes Toward and Experiences With Online Access to Scan Results

#### Theme 1: Greater Transparency and Patient Empowerment

Of the survey respondents who read their scan results online, 61% (22/36) thought it was an advantage that they could see these reports and 44% (16/36) felt it gave them more insight into and involvement in their illness.

Of the interview informants, 3 also felt they benefited from the online access through greater knowledge and insight into their individual disease. They experienced more shared medical decision making and could take a more active role in treatment issues. One informant described an improved collaboration with her physician with more effective and equal involvement:

I told them that I look up my record and prepare my appointment with the physician. Then they know exactly that they don’t have to tell me...this is what this means and what that means.... We can talk about the scan and the report and go on from there instead. And then she [the physician] can say “This is what I think we should do, what is your opinion about that?”,...and this means I get more involved in things.

Informant 2

#### Theme 2: Consequences of Bad News

In the survey, 35% (12/34) of respondents considered it a disadvantage to see the scan results before their hospital appointment due to the risk of reading bad news about disease progression or of misinterpreting the results. This issue was further explored in the interviews, where one informant who had previously been a diligent user of the PAEHR and had regularly read her scan results related an upsetting experience. She had read her scan result on a Friday afternoon and saw that it showed serious disease progression. She then had to spend the entire weekend with her family and the bad news, as she could not contact the hospital. Since then, she had changed her approach to only receiving information about her disease directly from her physician during hospital appointments.

#### Theme 3: Creation of a Dilemma

Two women described how online access gave them a dilemma of whether to read the report or to wait for the appointment at the hospital. As one respondent wrote in the questionnaire:

It is REALLY a dilemma!! My impatience to calm myself after a scan often drags me to look at the results on the online portal. But the problem is when it’s a “bad result,” the waiting time to my appointment at the hospital feels even longer and worse! I practice NOT looking up the scanning result online—but it’s difficult not to do it.

Respondent 8

#### Theme 4: Medical Terminology

In the survey, 32% (11/34) of respondents noted that the medical terminology used in scan results was a barrier to comprehension. In the interviews, the informants explained that they often used the built-in help functions in the PAEHR to look up medical terms and to see normal ranges for blood tests. However, it was often the overall meaning and consequences of the scan results that could be difficult to interpret, rather than individual medical terms. As one woman described it:

I know that progression means expansion, and I know that metastases are...when something is there.... But what does it mean if they are in three or four bones or just in one?...Because that was how it was at the next scan; what does that mean?

Informant 1

Two respondents to the survey suggested that if they could also view the images from their scans (which is currently not possible), they would have a better overview of their disease extent and development. This was also mentioned in the interviews, where several informants described how they found it difficult to get an overall picture of their disease status:

In a way, I feel like I’m missing that overview.... I think—well, they didn’t mention that in the report, so it’s probably gone...and it gives some insecurity...is it because they just didn’t see it this time or because it’s actually gone?...So if the report could be supported with some images, it would be fantastic.

Informant 3

#### Theme 5: Immediate Access Differs According to Scan Type

During the interviews, it became obvious that the speed of online access to scan results depended on whether patients were monitored with CT or PET/CT. Those having regular CT scans waited longer for their scan results to be released online, often until the day before their hospital appointment. In contrast, patients monitored with PET/CT could often read the scan result within 24 hours of their scan. They thus experienced a shorter waiting time but risked a longer period with frustration in the case of bad news or uncertain interpretation of the report. For the women monitored by CT, immediate online access was a more theoretical option than a reality, although they did not question this unless they had experienced a faster response time with a different examination.

#### Theme 6: Increased Need for Contact With the Oncology Team

Of the 15 women who used the immediate access to their scan results, 5 had experienced an urgent need to discuss the results with their oncologist. They had acted differently on this, either trying to phone the oncology department or their general practitioner, just waiting for their planned hospital appointment, or calling the diagnostic department to get more details about the scan results. The increased need for contact and reassurance from an oncologist was also clear in all 4 interviews. On weekdays, the patients could easily reach the oncology department, but it was a challenge outside general open hours.

#### Theme 7: Own Responsibility

All informants mentioned the risk of reading bad news, but none were in doubt that it was their own responsibility whether to access it or not. This came partly from the built-in informed consent process in the PAEHR system. As one woman said:

It’s my responsibility to log in, and it’s my responsibility to read the result.... There is a box to click where it asks “Are you sure you want to continue?”...and the preselected answer is NO....

Informant 1

All 4 informants were clear that they preferred to take this responsibility themselves and did not want to be spared or protected by the health care professionals:

It concerns the individual patient, it’s about the patient’s body, so why should this information be held back when it concerns the patient? Otherwise, it is up to them [the physicians] to sit and decide when you will get the information!

Informant 3

## Discussion

### Principal Findings

We believe this study is the first to focus on the patient perspective to online access to scan results. Previous authors have described online access as a doubled-edged sword with various challenges [8,13]. We present here some of the challenges that patients with metastatic breast cancer experience and what they consider to be the most important issues for the further development of an online patient record system.

Most of the women surveyed were aware of the online access opportunity but fewer than half read their scan results online. Most of them had a positive attitude toward online access, including prompt access to results. But some also indicated that prompt online access could create a dilemma about whether to look at the results and risk bad news, and could lead to greater need for contact with the oncology department. We found that the women who actively used the online access had a higher average level of education than nonusers.

### Knowledge and Use of Online Access to Scan Results

Although our sample comprised women with metastatic breast cancer in active follow-up with regular scans, and thus had high incentive to access their results, we found a smaller proportion of online users than expected. This was also lower than that reported from other studies among patients with cancer and chronic illness [4,7]. One reason may be that only one-third of the women surveyed had been informed about the online possibilities by a health care professional. The knowledge and attitudes of health care professionals are important for patient perceptions of the online patient record system and its successful implementation [6,13,17]. Health care professionals’ reluctance to make full online patient records accessible often originates from patients’ concerns, but it reduces the information level and use of digital possibilities for patients [5]. Health care professionals thus have an important role in educating and informing patients about online access.

### Attitudes Toward and Experiences With Online Access to Scan Results

The women in this study generally had a positive attitude toward online access to scan results. It gave them a chance to be better prepared for their appointment at the oncology department and thereby a feeling of equality and responsible involvement in their disease. Previous studies have shown that greater patient access to their own medical information can result in increased patient involvement and collaboration between patients and the health care team [4,5,8,18]. The participants found it positive that the anxious waiting time for scan results was shortened, although some felt it created a dilemma due to the risk of reading a negative or ambiguous result in the absence of a health professional. This is also an issue from the health care perspective, where the benefits of giving the patient a more active and informed role are offset by the risk of giving patients possibly upsetting information without input from a physician [3,13].

We further found that the patients were often challenged by the medical language used in the scan results, but in particular found it hard to understand the consequences of the results. Solutions have been suggested, such as an online dictionary [13]. An online dictionary is already available in the Danish online patient record system, however, and appears not to overcome all the difficulties that patients can have in understanding the scan results. The patients’ educational level and health literacy are important for their ability to interpret medical language [4,8], and this may be why we observed a higher educational level among active online users in this study.

### Variability of Timing of Access to Scan Results

It was clear from the interviews that the timing of online access to results differed according to whether patients were monitored with CT or PET/CT. This appeared to be due to different workloads and practices at the 2 diagnostic departments rather than the structure of the online records system. Although patients with delayed results had less of a dilemma in deciding whether to view a possibly discouraging result (as their hospital appointment was often the next day), we have to question whether these patients were given the same opportunities for participation and empowerment in their illness. An important aspect of the online patient record system is thus the different working practices at the hospital departments involved.

### Ethical Responsibility and the Patient’s Dilemma

Our results indicate that immediate access to test results was associated with both advantages and disadvantages, and that we need to increase awareness about maintaining optimal patient care in the digital health era. Previous studies have noted ethical challenges associated with giving patients prompt access to test results, especially in the diagnosis of cancer and its recurrence, and the increased need for urgent contact with the hospital [3,10,13].

The participants in our study desired full transparency and the opportunity to choose the amount of information they received. In an effort to minimize negative consequences, the Danish online patient record system has a built-in informed consent function that has the “No” response box preselected and informs the patient that they might view information that can be upsetting and ambiguous. Some health care sectors in other countries use different approaches, such as restricting the timing of posting online results in cases with sensitive diagnoses to ensure that a bad result can be given in person [3,10] or enabling patients to contact the oncology department through Web messaging [19].

Despite the participants’ concerns about immediate online access to results, only a few had experienced an urgent need to contact their physician after viewing the report online. Wiljer et al reported similar findings when investigating the support need among 250 patients with breast cancer [9]. However, more patients probably experienced this need but did not want to bother the hospital unnecessarily and thus waited for their planned appointment. Our study findings confirm that communication with the oncology department could be improved by a telephone hotline or a fast-response Web-message function.

### Strengths and Limitations

The mixed methods design of this study was an advantage, as it provided a more nuanced picture of the participants’ perspectives and experiences of online access to health records. The quantitative data gave an overview of the women’s use of, knowledge about, and attitudes toward online access, while the qualitative data went deeper and provided unique information about individual patient experiences, including new information about the differences in follow-up according to scan type. Although the input of patient representatives in designing the questionnaire ensured a patient-relevant perspective, we note the relatively small sample size for the survey and the use of partly open-ended questions. We interviewed only 4 informants, due to the exploratory design of the study. Because 4 interviews can be considered too few to achieve data saturation, we tried to accommodate this by including a specific patient group in the effort to decrease heterogeneity in the data. Furthermore, the interviews were conducted by a single person without prior interviewing experience. Although the external validity of the study must be considered to be low, as our self-selected participants with metastatic breast cancer may not represent the behavior and attitudes of cancer patients in general, the results indicate issues that are likely to be important aspects of any online system that gives patients access to their health records.

### Conclusion

The patients with metastatic breast cancer who participated in this study generally had a positive attitude toward electronic access to their scan results, and those who used the online access played a greater participatory role in their disease and its management. We noted some challenges, however, including the patients’ dilemma of whether to view results that might cause distress in the absence of information and interpretation by a health professional. It could also be difficult for the women to understand the consequences of the results for their individual treatment plan.

The study findings suggest that immediate online access to scan results should be available to patients, but it needs a support function alongside that ensures optimal patient care. As the participants who actively used the online health record system to view their results were generally more highly educated than nonusers, we suggest that health professionals take a more active role in informing a wider patient group about the digital possibilities. This should be followed up with further studies monitoring patients’ experiences with online access and their needs for supplementary contact or information.

## Abbreviations

CT: computed tomography
PAEHR: patient-accessible electronic health record
PET/CT: positron emission tomography with computed tomography

## References

[1] Nguyen L, Bellucci E, Nguyen LT (2014). Electronic health records implementation: an evaluation of information system impact and contingency factors. Int J Med Inform.

[2] Brodersen S, Lindegaard H (2015). Empowering patients through healthcare technology and information? The challenge of becoming a Patient 2.0. Int J Health Tech Manag.

[3] Halamka JD, Mandl KD, Tang PC (2008). Early experiences with personal health records. J Am Med Inform Assoc.

[4] Gerber DE, Laccetti AL, Chen B, Yan J, Cai J, Gates S, Xie Y, Lee SJC (2014). Predictors and intensity of online access to electronic medical records among patients with cancer. J Oncol Pract.

[5] Woods SS, Schwartz E, Tuepker A, Press NA, Nazi KM, Turvey CL, Nichol WP (2013). Patient experiences with full electronic access to health records and clinical notes through the My HealtheVet Personal Health Record Pilot: qualitative study. J Med Internet Res.

[6] Bos L, Marsh AJ, Carroll D, Gupta S, Rees M (2008). Patient 2.0 empowerment.

[7] Andreassen HK, Bujnowska-Fedak MM, Chronaki CE, Dumitru RC, Pudule I, Santana S, Voss H, Wynn R (2007). European citizens' use of E-health services: a study of seven countries. BMC Public Health.

[8] Keselman A, Slaughter L, Smith CA, Kim H, Divita G, Browne A, Tsai C, Zeng-Treitler Q (2007). Towards consumer-friendly PHRs: patients' experience with reviewing their health records. AMIA Annu Symp Proc.

[9] Wiljer D, Urowitz S, Apatu E, Leonard K, Quartey NK, Catton P (2010). Understanding the support needs of patients accessing test results online. PHRs offer great promise, but support issues must be addressed to ensure appropriate access. J Healthc Inf Manag.

[10] Beard L, Schein R, Morra D, Wilson K, Keelan J (2012). The challenges in making electronic health records accessible to patients. J Am Med Inform Assoc.

[11] (2020). Din sundhedsportal.

[12] sundhed.dk (2016). Strategi for sundhed.dk 2016-2018.

[13] Wiljer D, Urowitz S, Apatu E, DeLenardo C, Eysenbach G, Harth T, Pai H, Leonard KJ, Canadian Committee for Patient Accessible Health Records (2008). Patient accessible electronic health records: exploring recommendations for successful implementation strategies. J Med Internet Res.

[14] Wiljer D, Leonard KJ, Urowitz S, Apatu E, Massey C, Quartey NK, Catton P (2010). The anxious wait: assessing the impact of patient accessible EHRs for breast cancer patients. BMC Med Inform Decis Mak.

[15] Smith JA (2008). Qualitative Psychology: A Practical Guide to Research Methods. 2nd edition.

[16] Biggerstaff D, Thompson AR (2008). Interpretative phenomenological analysis (IPA): a qualitative methodology of choice in healthcare research. Qual Res Psychol.

[17] Smith SK, Dixon A, Trevena L, Nutbeam D, McCaffery KJ (2009). Exploring patient involvement in healthcare decision making across different education and functional health literacy groups. Soc Sci Med.

[18] Tang PC, Ash JS, Bates DW, Overhage JM, Sands DZ (2006). Personal health records: definitions, benefits, and strategies for overcoming barriers to adoption. J Am Med Inform Assoc.

[19] Hassol A, Walker JM, Kidder D, Rokita K, Young D, Pierdon S, Deitz D, Kuck S, Ortiz E (2004). Patient experiences and attitudes about access to a patient electronic health care record and linked web messaging. J Am Med Inform Assoc.

